# Association between Osteoporosis and Low Hemoglobin Levels: A Nested Case–Control Study Using a National Health Screening Cohort

**DOI:** 10.3390/ijerph18168598

**Published:** 2021-08-14

**Authors:** So-Young Kim, Dae-Myoung Yoo, Chanyang Min, Hyo-Geun Choi

**Affiliations:** 1 Department of Otorhinolaryngology-Head & Neck Surgery, CHA Bundang Medical Center, CHA University, Seongnam 13496, Korea; sossi81@hanmail.net; 2 Hallym Data Science Laboratory, Hallym University College of Medicine, Anyang 14068, Korea; ydm1285@naver.com (D.-M.Y.); joicemin@naver.com (C.M.); 3 Graduate School of Public Health, Seoul National University, Seoul 08826, Korea; 4 Department of Otorhinolaryngology-Head & Neck Surgery, Hallym University College of Medicine, Anyang 14068, Korea

**Keywords:** osteoporosis, hemoglobin, anemia, case–control studies, cohort studies

## Abstract

An association between anemia and an increased risk of osteoporosis has been suggested. The goal of this study was to estimate the association of hemoglobin (Hb) level with osteoporosis. A total of 69,760 osteoporosis patients aged ≥ 40 years old from the Korean National Health Insurance Service Health Screening Cohort were enrolled. From an identical cohort database, 69,760 comparison participants were randomly selected. Hb levels before the onset of osteoporosis were evaluated. The association of Hb level with osteoporosis was analyzed using a conditional logistic regression model adjusted for obesity, smoking status, alcohol consumption, systolic blood pressure, diastolic blood pressure, fasting blood glucose, total cholesterol, and the Charlson comorbidity index score. Fifteen percent of the osteoporosis group and 14.17% of the comparison group had anemia. The Hb level was associated with 0.98-fold lower odds for osteoporosis (95% confidence intervals = 0.97–0.99, *p* < 0.001). A low Hb level was associated with a high risk of osteoporosis in the adult population. There was a consistent association between a low Hb level and osteoporosis in patients with comorbidities.

## 1. Introduction

Osteoporosis is often diagnosed on the basis of low bone mineral density (BMD). In addition to a low body mass index (BMI), an increased risk of fracture is included in the diagnosis of osteoporosis. The prevalence of osteoporosis has been estimated to be approximately 40% in white postmenopausal women [1,2,3,4]. Low BMD is often ignored until fracture or other complications occur. Thus, the predictive factors for fracture risk and osteoporosis have been investigated by many researchers. Low BMD has been reported to be associated with aging, low body weight, physical inactivity, smoking, excessive alcohol consumption, diabetes, dyslipidemia, and pernicious anemia [5,6,7]. In addition, chronic iron-deficiency anemia has been suggested to induce bone resorption and increase the risk of osteoporosis [8].

Anemia is a condition characterized by a low hemoglobin (Hb) level. The prevalence of anemia was estimated to be approximately 2.5% in men and 9.0% in women in the 50–59-year-old Korean population [9]. The most common type of anemic condition is microcytic anemia, which is caused by a number of conditions with reduced production of Hb; microcytic anemia includes thalassemia, anemia of chronic inflammation, and iron-deficiency anemia [10]. As with osteoporosis, anemia may go underdiagnosed or unrecognized until clinical manifestation such as dizziness or facial pallor development. The risk factors for anemia include nutritional deficiency, infection, and blood loss, including blood loss during menstruation period and chronic inflammation [10,11]. Anemia and osteoporosis share common risk factors associated with nutritional deficiency and inflammation, and both have female predominance.

We postulated that preceding anemia may induce osteoporosis. Most previous studies evaluated the association of osteoporosis with specific types of anemia, including iron-deficiency anemia [12], sickle cell anemia [13], and thalassemia [14], or limited the study cohort to specific populations such as elderly individuals [11] or postmenopausal women [15]. A cross-sectional study reported the association between Hb levels and BMD in an age- and sex-specific manner [16]. That study showed a positive association between the Hb level and BMD in men and postmenopausal women [16]; however, anemic patients had different age, sex, income, and region of residence distributions, as well as different medical histories [16]. To overcome these limitations, we recruited a large cohort population and a matched comparison group. Moreover, to identify temporal associations between anemia and osteoporosis, a previous history of anemia before the diagnosis of osteoporosis was analyzed. Using a large study population, this study was aimed to evaluate the impact of the prior presence of anemia on the occurrence of osteoporosis in an adult population.

## 2. Materials and Methods

### 2.1. Study Population

This is a nested case–control study using a national health screening cohort. The Korean National Health Insurance Service Health Screening Cohort data over the period from 2002 to 2013 were used. The health check-up data of all Koreans ≥ 40 years old and the health claim data from the Korean Health Insurance Review and Assessment system were analyzed. A detailed description of the Korean National Health Insurance Service Health Screening Cohort data is presented elsewhere [17].

### 2.2. Definition of Osteoporosis

Osteoporosis was defined as ≥2 relevant International Classification of Diseases, 10th revision (ICD-10) codes (M80-M82: osteoporosis with pathological fracture, osteoporosis without pathological fracture, and osteoporosis in diseases classified elsewhere). Among the identified patients, those who had been tested for bone density using X-ray or computed tomography (CT) were included following the methods in our previous study [18].

### 2.3. Hemoglobin Concentration

The current Hb concentration was calculated before the day of diagnosis of osteoporosis (index date). The Hb concentration was measured from a venous blood sample using an automated hematology analyzer. In men, anemia severity (hemoglobin, g/dL) was categorized as ≥13 (normal), <13 to ≥11 (mild), <11 to ≥8 (moderate), and <8 (severe). In women, anemia severity (hemoglobin) was categorized as ≥12 (normal), <12 to ≥11 (mild), <11 to ≥8 (moderate), and <8 (severe).

### 2.4. Participant Selection

**Figure 1:**
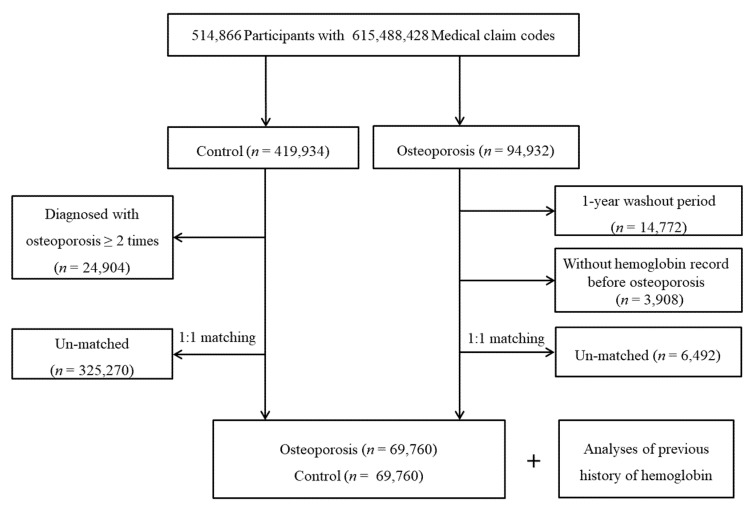
A schematic illustration of the participant selection process that was used in the present study. Of a total of 514,866 participants, 69,760 osteoporosis patients were 1:1 matched with 69,760 comparison participants for age, sex, income, and region of residence.

Osteoporosis patients were selected from 514,866 participants with 615,488,428 medical claim codes from 2002 through 2015 (*n* = 94,932) following our definition of osteoporosis. To ensure that osteoporosis was newly diagnosed, we excluded osteoporosis patients who were treated for osteoporosis in 2002 (*n* = 14,772), leaving only osteoporosis patients who were diagnosed in 2003 or later. The first osteoporosis treatment date was set as the index date. Among the osteoporosis patients, we excluded those who did not have Hb records before the index date (*n* = 3908). The participants who were not included in the osteoporosis group were allocated to the comparison group (*n* = 419,934). Among them, participants who had a history of osteoporosis diagnosis without a bone density test were excluded (*n* = 24,904). Osteoporosis patients were 1:1 matched with comparison participants for age, sex, income, and region of residence. To minimize selection bias, the comparison participants were selected with the random number method. To ensure the same index date for each osteoporosis patient and their matched comparison participant, the index date of the comparison participant was set as the index date of their matched osteoporosis patient. During the matching procedure, 6492 osteoporosis patients and 325,270 comparison participants were excluded. Ultimately, 69,760 osteoporosis patients were 1:1 matched with 69,760 comparison participants (Figure 1).

### 2.5. Covariates

Age groups were divided into 5-year intervals: 40–44, 45–49, 50–54, …, and 85+ years old. A total of 10 age groups were specified. Income groups were classified into 5 classes (class 1 (lowest income) to class 5 (highest income)). The region of residence was grouped into urban and rural areas following our previous study [17,19].

Tobacco smoking and alcohol consumption were categorized following a previous study (nonsmoker, past smoker, and current smoker) [17]. Past smoker was defined as the participants who quit smoking more than 1 year prior. Obesity was classified using BMI (kg/m^2^) based on the Asia-Pacific criteria [20]. Systolic blood pressure, diastolic blood pressure, fasting blood glucose, and total cholesterol were measured. Missing BMI (109/139,520 (0.078%)), systolic blood pressure (65/139,520 (0.047%)), diastolic blood pressure (78/139,520 (0.056%)), fasting blood glucose (39/139,520 (0.028%)), and total cholesterol (106/139,520 (0.076%)) data were replaced by the mean values of each variable from the final selected participants. The participants received a Charlson comorbidity index (CCI) score from 0 (no comorbidities) to 29 (multiple comorbidities) according to their comorbidities [21].

### 2.6. Statistical Analyses

The general characteristics were compared between the osteoporosis and comparison groups using the chi-square test.

To analyze the odds ratios (ORs) with 95% confidence intervals (CIs), a conditional logistic regression model for osteoporosis associated with Hb level was calculated. Crude and adjusted models were constructed. In the adjusted model, obesity, smoking, alcohol consumption, systolic blood pressure, diastolic blood pressure, fasting blood glucose, total cholesterol, and CCI scores were considered covariates. The analyses were stratified by age, sex, income, and region of residence.

For subgroup analysis, we classified participants by age and sex (<65 years old and ≥65 years old; men and women) and analyzed crude and adjusted models.

Additionally, we analyzed other subgroups. We classified participants by obesity status (BMI < 23 or BMI ≥ 23), smoking status (nonsmoker or smoker), alcohol consumption (<1 time a week or ≥1 time a week), blood pressure (normal or hypertension (systolic blood pressure ≥ 120 mmHg or diastolic blood pressure ≥ 80 mmHg)), fasting blood glucose (normal (<100 mg/dL) or hyperglycemia (≥100 mg/dL)), and total cholesterol (normal (<200 mg/dL) or dyslipidemia (≥200 mg/dL)) [22,23,24]. An unconditional logistic regression model for osteoporosis associated with Hb level was constructed to analyze the ORs with 95% CIs.

Two-tailed analyses were performed, and significance was defined as *p* values less than 0.05. SAS version 9.4 (SAS Institute Inc., Cary, NC, USA) was used for the statistical analyses.

### 2.7. Ethical Considerations

The ethics committee of Hallym University (23 October 2019) permitted this study. Written informed consent was waived by the Institutional Review Board.

## 3. Results

In total, 15.15% (10,563/69,760) and 14.17% (9882/69,760) of the osteoporosis and comparison groups, respectively, had a history of anemia (*p* < 0.001, Table 1). In detail, 11.8% (8256/69,760), 3.2% (2223/69,760), and 0.1% (84/69,760) of the osteoporosis group had mild, moderate, and severe anemia, respectively. The osteoporosis group had higher prevalence rates of underweight; nonsmoking; infrequent alcohol consumption; and normal blood pressure, fasting blood glucose, and total cholesterol levels than the comparison group (all *p* < 0.001). The CCI scores were different between the osteoporosis and comparison groups (*p* < 0.001).

A high Hb level was associated with lower odds of osteoporosis (adjusted OR (aOR) = 0.98, 95% CI = 0.97–0.99, *p* < 0.001, Table 2). There were inverse associations of Hb level with osteoporosis in males < 65 years old, males ≥ 65 years old, and females ≥ 65 years old (aOR = 0.87, 95% CI = 0.83–0.92, *p* < 0.001 for <65-year-old males; aOR = 0.94, 95% CI = 0.92–0.97, *p* < 0.001 for ≥65-year-old males; and aOR = 0.98, 95% CI = 0.96–0.99, *p* = 0.011 for ≥65-year-old females).

In addition, the obesity (BMI ≥ 23), past smoker, current smoker, infrequent alcohol consumption, high blood pressure, high fasting blood glucose, and high total cholesterol groups had lower odds of osteoporosis associated with high Hb levels than their counterparts (Table S1).

## 4. Discussion

Low Hb levels were associated with increased odds of osteoporosis in the adult population. The association of low Hb levels with osteoporosis was verified in the model adjusted for many potential confounders, including obesity, smoking, alcohol consumption, laboratory findings, and comorbidities. Moreover, among participants with comorbid conditions, such as obesity, smoking, hypertension, diabetes, and dyslipidemia, there was a consistent association of low Hb levels with osteoporosis.

Some previous studies proposed the association of anemia with osteoporosis.

In a cross-sectional study, BMD and Hb levels showed a positive association in adult men (β-coefficient ± standard error = 0.117 ± 0.004, *p* = 0.015) [16]. Another cross-sectional study described the association of low BMD with low Hb levels and the presence of anemia in postmenopausal women (aOR of low BMD for anemia = 2.483, 95% CI = 1.309–4.712, *p* = 0.005) [15]. There was also a positive correlation between Hb level and BMD in the population aged 75 years and older (β-coefficient = 0.13, 95% CI = 0.01–0.25, *p* = 0.030) [25]. A longitudinal study demonstrated an 84% higher risk of osteoporosis in iron-deficiency anemia patients (95% CI = 1.67–1.97) [12].

Elevated osteoclast activity and subsequent accelerated bone resorption have been proposed as underlying mechanisms of osteoporosis in anemia patients. The reduced blood volume stimulates the proliferation of hematopoietic cells, including osteoclasts [26]. Proliferated osteoclasts may stimulate bone resorption. Although osteoblast formation can also be stimulated by blood loss, stimulated bone resorption may hinder bone remodeling cycles, which might result in osteoblast fatigue [26].

Hypoxemia in anemic patients may mediate the risk of osteoporosis. Chronic hypoxia may increase oxidative stress, and acidification of the extracellular matrix can impair bone metabolism [27,28]. Hypoxia is presumed to diminish the growth of osteoblasts. Hypoxic conditions have been shown to reduce BMD in both humans and rats [29]. The presence of chronic diseases could accentuate the impact of an anemic state on osteoporosis [27]. Several chronic diseases related to hypoxia, such as chronic obstructive pulmonary disease (COPD), obstructive sleep apnea (OSA), and left ventricular dysfunction, have been shown to be related to reduced BMD [29,30]. In a previous study, BMD was correlated with the severity of airway obstruction measured by pulmonary function tests and arterial blood pH in COPD patients, although no association between BMD and the presence of COPD was noted [28].

According to the subgroup analyses, there was a negative association between Hb levels and osteoporosis in patients with comorbidities. The relatively high susceptibility to hypoxia and pre-existing inflammation might potentiate the association of anemia with osteoporosis in these patients [31]. Several previous studies reported an association of anemia with osteoporosis in patients with COPD or renal failure requiring hemodialysis [32,33]. There was no association of anemia with osteoporosis in middle-aged women in this study. This might be due to different etiologies of anemia in this female population of reproductive age. Because of menstrual cycles and pregnancy, iron-deficiency anemia due to blood loss is common in middle-aged women. Thus, chronic hypoxia and inflammation might be less common, and the impact of anemia on osteoporosis may not be considerable in this group. In line with our results, a previous study reported no or an inverse association of Hb levels with BMD in premenopausal women [16].

This study obtained data from a large nationwide cohort. Age, sex, income, and region of residence were similar between the osteoporosis and comparison groups, which were randomly matched. Anemia was defined based on the Hb level, which improved the diagnostic accuracy. There was no significant association between osteoporosis and anemia when anemia was binarily defined based on Hb levels. However, there was a significant association between osteoporosis and Hb levels, although the ORs (per 1 g/dL change in Hb level) were not considerably high in the present results. Additionally, data on BMD in osteoporosis patients were unavailable. The types of anemia, such as iron-deficiency anemia, sickle cell anemia, thalassemia, and Fanconi anemia, could not be differentiated. Moreover, the prescription histories for anemia and osteoporosis patients were not available in the Korean National Health Insurance Service Health Screening Cohort database. Although many potential confounders were considered, the possible impacts of physical activity, nutritional intake, and steroid medication use could not be excluded in the current study.

## 5. Conclusions

Low Hb levels were negatively associated with osteoporosis in the adult population. A protective effect of high Hb on osteoporosis was noted in participants with comorbidities. The correction of low Hb could have a protective role to prevent the occurrence of osteoporosis, especially in the population with comorbidities.

## Figures and Tables

**Table 1:** General characteristics of participants.

Characteristics		Total Participants		
		Osteoporosis (*n*, %)	Control (*n*, %)	*p*-Value
Age (years old)				1.000
	40–44	728 (1.0)	728 (1.0)	
	45–49	4110 (5.9)	4110 (5.9)	
	50–54	9401 (13.5)	9401 (13.5)	
	55–59	10,982 (15.7)	10,982 (15.7)	
	60–64	12,559 (18.0)	12,559 (18.0)	
	65–69	15,698 (22.5)	15,698 (22.5)	
	70–74	8874 (12.7)	8874 (12.7)	
	75–79	5021 (7.2)	5021 (7.2)	
	80–84	2006 (2.9)	2006 (2.9)	
	85+	381 (0.6)	381 (0.6)	
Sex				1.000
	Male	9231 (13.2)	9231 (13.2)	
	Female	60,529 (86.8)	60,529 (86.8)	
Income				1.000
	1 (lowest)	13,281 (19.0)	13,281 (19.0)	
	2	10,196 (14.6)	10,196 (14.6)	
	3	11,067 (15.9)	11,067 (15.9)	
	4	14,217 (20.4)	14,217 (20.4)	
	5 (highest)	20,999 (30.1)	20,999 (30.1)	
Region of residence				1.000
	Urban	28,090 (40.3)	28,090 (40.3)	
	Rural	41,670 (59.7)	41,670 (59.7)	
Obesity ^†^				<0.001 *
	Underweight	2282 (3.3)	1687 (2.4)	
	Normal	26,402 (37.9)	23,210 (33.3)	
	Overweight	18,257 (26.2)	18,175 (26.1)	
	Obese I	20,749 (29.7)	23,565 (33.8)	
	Obese II	2070 (3.0)	3123 (4.5)	
Smoking status				<0.001 *
	Nonsmoker	63,724 (91.4)	63,273 (90.7)	
	Past smoker	2449 (3.5)	2454 (3.5)	
	Current smoker	3587 (5.1)	4033 (5.8)	
Alcohol consumption				<0.001 *
	<1 time a week	61,462 (88.1)	61,021 (87.5)	
	≥1 time a week	8298 (11.9)	8739 (12.5)	
Systolic blood pressure				<0.001 *
	<120 mmHg	21,374 (30.6)	19,277 (27.6)	
	120–139 mmHg	31,510 (45.2)	30,978 (44.4)	
	≥140 mmHg	16,876 (24.2)	19,505 (28.0)	
Diastolic blood pressure				<0.001 *
	<80 mmHg	33,298 (47.7)	30,735 (44.1)	
	80–89 mmHg	23,626 (33.9)	24,121 (34.6)	
	≥90 mmHg	12,836 (18.4)	14,904 (21.4)	
Fasting blood glucose				<0.001 *
	<100 mg/dL	48,016 (68.8)	44,884 (64.3)	
	100–125 mg/dL	16,988 (24.4)	18,004 (25.8)	
	≥126 mg/dL	4756 (6.8)	6872 (9.9)	
Total cholesterol				<0.001 *
	<200 mg/dL	33,337 (47.8)	32,716 (46.9)	
	200–239 mg/dL	24,819 (35.6)	24,575 (35.2)	
	≥240 mg/dL	11,604 (16.6)	12,469 (17.9)	
CCI score				
	0	43,316 (62.1)	45,502 (65.2)	<0.001 *
	1	11,832 (17.0)	10,305 (14.8)	
	2	6697 (9.6)	5890 (8.4)	
	3	3500 (5.0)	3229 (4.6)	
	≥4	4415 (6.3)	4834 (6.9)	
Anemia severity ^‡^				<0.001 *
	Normal	59,197 (84.9)	59,878 (85.8)	
	Mild	8256 (11.8)	7508 (10.8)	
	Moderate	2223 (3.2)	2249 (3.2)	
	Severe	84 (0.1)	125 (0.2)	

Abbreviations: CCI, Charlson comorbidity index. * Chi-square test. Significance at *p* < 0.05. ^†^ Obesity (BMI, body mass index, kg/m^2^) was categorized as <18.5 (underweight), ≥18.5 to <23 (normal), ≥23 to <25 (overweight), ≥25 to <30 (obese I), and ≥30 (obese II). ^‡^ In men, anemia severity (hemoglobin, g/dL) was categorized as ≥13 (normal), <13 to ≥11 (mild), <11 to ≥8 (moderate), and <8 (severe). In women, anemia severity (hemoglobin) was categorized as ≥12 (normal), <12 to ≥11 (mild), <11 to ≥8 (moderate), and <8 (severe).

**Table 2:** Crude and adjusted odds ratios (95% confidence interval) for osteoporosis in hemoglobin according to age and sex.

Characteristics		Odds Ratios for Osteoporosis			
		Crude ^†^	*p*-Value	Adjusted ^†,‡^	*p*-Value
Total participants (*n* = 139,520)					
	Hemoglobin	0.96 (0.95–0.97)	<0.001 *	0.98 (0.97–0.99)	<0.001 *
Age < 65 years old, men (*n* = 4588)					
	Hemoglobin	0.82 (0.78–0.87)	<0.001 *	0.87 (0.83–0.92)	<0.001 *
Age < 65 years old, women (*n* = 70,972)					
	Hemoglobin	0.98 (0.97–0.99)	0.002 *	1.01 (0.99–1.02)	0.355
Age ≥ 65 years old, men (*n* = 13,874)					
	Hemoglobin	0.91 (0.89–0.94)	<0.001 *	0.94 (0.92–0.97)	<0.001 *
Age ≥ 65 years old, women (*n* = 50,086)					
	Hemoglobin	0.96 (0.94–0.97)	<0.001 *	0.98 (0.96–0.99)	0.011 *

Abbreviation: CCI, Charlson comorbidity index. * Conditional logistic regression model, significance at *p* < 0.05. ^†^ Models stratified by age, sex, income, and region of residence. ^‡^ A model adjusted for obesity, smoking, alcohol consumption, systolic blood pressure, diastolic blood pressure, fasting blood glucose, total cholesterol, and CCI scores.

## Data Availability

Releasing of the data by the researcher is not allowed legally. All data are available from the database of the Korea Centers for Disease Control and Prevention. The Korea Centers for Disease Control and Prevention allows data access, at a particular cost, for any researcher who promises to follow the research ethics. Data of this article can be downloaded from the website after promising to follow the research ethics.

## Supplementary Materials

The following are available online at https://www.mdpi.com/article/10.3390/ijerph18168598/s1, Table S1 Subgroup analyses of crude and adjusted odds ratios (95% confidence interval) for osteoporosis in hemoglobin according to obesity, smoking, alcohol consumption, blood pressure, fasting blood glucose, and total cholesterol.
