# Supplementary material for: Association between Osteoporosis and Low Hemoglobin Levels: A Nested Case–Control Study Using a National Health Screening Cohort
Source: Int J Environ Res Public Health. 2021 Aug 14;18(16):8598. doi: 10.3390/ijerph18168598 (PMC8394089; doi:10.3390/ijerph18168598)
Supplement: Supplementary file 1 [file ijerph-18-08598-s001.zip › ijerph-1336068-supplementary.pdf]

**Table S1.** Subgroup analyses of crude and adjusted odd ratios (95% confidence interval) for osteoporosis in hemoglobin according to obesity, smoking, alcohol consumption, blood pressure, fasting blood glucose, and total cholesterol

| Characteristics | Odds ratios for osteoporosis | | | |
|---|---|---|---|---|
| | Crude | P-value | Adjusted† | P-value |
| BMI < 23 (n = 53,581) | | | | |
| Hemoglobin | 0.99 (0.98-1.01) | 0.340 | 1.00 (0.98-1.01) | 0.575 |
| BMI ≥ 23 (n = 85,939) | | | | |
| Hemoglobin | 0.96 (0.95-0.97) | <0.001* | 0.97 (0.96-0.98) | <0.001* |
| Nonsmoker (n = 126,997) | | | | |
| Hemoglobin | 0.97 (0.96-0.98) | <0.001* | 0.99 (0.98-1.00) | 0.088 |
| Past smoker and current smoker (n = 12,523) | | | | |
| Hemoglobin | 0.95 (0.93-0.98) | <0.001* | 0.94 (0.91-0.96) | <0.001* |
| Alcohol consumption < 1 time a week (n = 122,483) | | | | |
| Hemoglobin | 0.97 (0.96-0.98) | <0.001* | 0.99 (0.98-1.00) | 0.016* |
| Alcohol consumption ≥ 1 time a week (n = 17,037) | | | | |
| Hemoglobin | 0.95 (0.93-0.98) | <0.001* | 0.98 (0.95-1.00) | 0.069 |
| Systolic blood pressure < 140 mmHg and diastolic blood pressure < 90 mmHg (n = 97,314) | | | | |
| Hemoglobin | 0.97 (0.96-0.98) | <0.001* | 0.99 (0.98-1.00) | 0.029* |
| Systolic blood pressure ≥ 140 mmHg or diastolic blood pressure ≥ 90 mmHg (n = 42,206) | | | | |
| Hemoglobin | 0.96 (0.94-0.97) | <0.001* | 0.98 (0.96-0.99) | 0.004* |
| Fasting blood glucose < 100 mg/dL (n = 92,900) | | | | |
| Hemoglobin | 0.98 (0.97-0.99) | <0.001* | 1.00 (0.99-1.01) | 0.693 |
| Fasting blood glucose ≥ 100 mg/dL (n = 46,620) | | | | |
| Hemoglobin | 0.94 (0.93-0.96) | <0.001* | 0.96 (0.95-0.98) | <0.001* |

| | | | | |
|---|---|---|---|---|
| Total cholesterol < 200 mg/dL (n = 66,053) | | | | |
| Hemoglobin | 0.97 (0.96-0.98) | <0.001* | 0.99 (0.97-1.00) | 0.053 |
| Total cholesterol ≥ 200 mg/dL (n = 73,467) | | | | |
| Hemoglobin | 0.96 (0.95-0.97) | <0.001* | 0.98 (0.97-1.00) | 0.015* |

Abbreviation: CCI, Charlson Comorbidity Index

* Logistic regression model, Significance at $P < 0.05$

† A model adjusted for age, sex, income, region of residence, obesity, smoking, alcohol consumption, systolic blood pressure, diastolic blood pressure, fasting blood glucose, total cholesterol, and CCI scores.
